# OrthoGrafter: Rapid Identification of Orthologs from Precomputed Placement in Phylogenetic Trees

**DOI:** 10.1007/s00239-025-10279-z

**Published:** 2025-11-22

**Authors:** Christopher M. Williams, Paul D. Thomas

**Affiliations:** Department of Population and Public Health Sciences, University of Southern California, Los Angeles, CA USA

**Keywords:** Phylogenetics, Orthologs, Homology

## Abstract

The identification of orthologs plays an important role in comparative genomics and function inference. Here, we present OrthoGrafter, a bioinformatics tool for taking one or more query sequences and inferring a set of orthologs drawn from the collection of 143 well annotated species in the PANTHER database of reconciled gene trees. OrthoGrafter takes sets of graft points output by the highly used TreeGrafter software (either the standalone package or InterProScan), and for each one, outputs a list of predicted orthologous genes from the grafted PANTHER tree (with the ability to additionally output paralog and xenolog sets). If the taxonomic identifier for the query is also provided, OrthoGrafter incorporates the novel step of adjusting the graft point to facilitate consistent taxonomic assignment for the graft within the reconciled gene family, which we demonstrate shows an improvement in the ortholog inference via correlation with orthologs provided by the OMA database. Lightweight and utilizing precomputed results to enable rapid determination of ortholog predictions for large sample groups, OrthoGrafter is available at https://github.com/pantherdb/OrthoGrafter..

**Supplementary Information:**

The online version of this article contains supplementary material available 10.1007/s00239-025-10279-z.

## Introduction

Homologous genes represent pairs of genes that have descended from the same ancestral sequence. Orthologs represent a subtype of homologous genes which diverged due to a speciation event, as opposed to other types of evolutionary events such as duplication (leading to paralogs) or horizontal transfer (when a gene is copied from one organism into another, leading to xenologs if they occurred anywhere on the path between two genes) (Koonin 2005). The identification of orthologous genes represents a significant area of biological research, as their identification can aid in both understanding the evolutionary history of gene families, as well as in the determination of protein functional annotations, which in turn could assist in real world applications such as drug discovery (Jain et al. 2017). A number of methods have been developed to infer orthologs, many of which have been benchmarked against a standard set of tests (Altenhoff et al. 2024a, 2016). Orthologs among the set of 78 genomes in this benchmark set are publicly available for all benchmarked methods (Altenhoff et al. 2016). In addition, many of these methods have been applied to a large number of other genomes, and pre-calculated results are available for download. For other genomes, software packages for ortholog inference can be downloaded and run locally.

This paper describes OrthoGrafter, a lightweight, efficient software package, enabling the rapid determination of well annotated ortholog sets from the set of PANTHER gene families. OrthoGrafter builds upon the work of TreeGrafter (Tang et al. 2019), utilizing the graft positions identified by TreeGrafter.

PANTHER gene families are a set of reconciled gene trees, in which leaf nodes represent extant genes sequences, and internal nodes the points of evolutionary divergence. Internal nodes have labels for both the evolutionary event they represent - speciation, duplication or horizontal transfer, and their clade/taxon (for speciation nodes specifically). PANTHER trees are phylogenetic trees reconciled to the species tree such that they are taxonomically consistent, with all descendant speciation nodes of an ancestral speciation node also being its taxonomic descendants. The one exception to this is if there is a horizontal transfer between two speciation nodes, in which case they may not be taxonomically consistent. PANTHER trees were constructed using the GIGA algorithm (Thomas 2010; Thomas et al. 2022). PANTHER 17, the current PANTHER version utilized by OrthoGrafter, contains 15,619 gene families with genes from 143 genomes spanning the tree of life, and includes all well-studied genomes such as those from model organisms. It also contains branches lengths (based on the number of amino acid substitutions per site) as a of measure divergence between nodes (Thomas et al. 2022).

TreeGrafter is a program which when provided with a gene sequence, utilizes Hidden Markov Models to identify the best fitting PANTHER gene tree and branch within the tree along which the input sequence diverged, outputting a graft location (a combination of PANTHER family and node identifier within the family) (Thomas et al. 2022; Tang et al. 2019). In this way TreeGrafter attempts to place query sequences in their evolutionary context with respect to the set of sequences incorporated in the PANTHER phylogenetic gene trees. Precomputed TreeGrafter graft locations are available for proteins in the UniProt database (see Supplementary Table 1). For sequences not available in UniProt, one may either run the standalone TreeGrafter software, or InterProScan (Blum et al. 2024), which offers a modified implementation of TreeGrafter.

A significant drawback of TreeGrafter however, is that its graft locations are based only on sequence information (using maximum parsimony), and they are often not consistent with the taxonomy of species from which the input sequence was derived. While PANTHER families are reconciled gene trees, with their genes mapped to the species tree (Thomas et al. 2022), TreeGrafter graft locations are not reconciled. Being reconciled is important because it enables more accurate prediction of the correct set of ancestors and thus orthologs in a tree based on shared divergence from ancestral speciation nodes. Because TreeGrafter grafts are not reconciled this means ortholog sets predicted based on these graft points can be less informative. OrthoGrafter addresses this limitation.

## Methods

OrthoGrafter utilize sample inputs and implicit and explicit taxonomic data to update the graft positions. Samples are input with information on their species and TreeGrafter graft locations (the PANTHER tree and graft node ID). The species is used to identify each inputs relevant taxonomic information, which includes both explicit taxon information, ie the taxons which match those labeled to internal speciation nodes in PANTHER, and implicit taxon information, ie the taxons which exist along branches between the explicit taxons, which are not labeled in PANTHER (due to no divergences occurring in PANTHER at those taxons). Using this information OrthoGrafter attempts to update the graft locations to reconcile them within their PANTHER families, and then identifies the ortholog sets for each sample. This is a similar workflow to software packages such as SHOOT (Emms and Kelly 2022), except that it leverages the phylogenetic trees available from the PANTHER resource, and the highly used TreeGrafter (and InterProScan) software packages, for the most compute-intensive steps. Like SHOOT, which uses OrthoFinder, OrthoGrafter, which uses PANTHER, computes orthologs using a method that has been extensively benchmarked and characterized for accuracy (Altenhoff et al. 2024a, 2016).

### Algorithm Steps

In brief the algorithm is provided with a set of inputs which contain information on the species ID, and graft locations (ie the PANTHER tree and node ID). It then tries to update the graft location by predicting where it actually evolutionary diverged in the phylogenetic gene tree. It checks first if any descendant nodes are a good fit, before checking ancestor and sibling nodes in the tree. Below is a general explanation of the steps utilized in the algorithms function:

1. **Determine Ancestral-Taxons** The set of ancestral taxons for each input sample is determined using NCBI taxonomic relationships from the NCBI ontology (Federhen 2003), supplemented by a set of unique PANTHER-specific taxonomic IDs (to address differences between PANTHER clades and the NCBI taxonomy).
2. **Identify Descendant-Node Ancestral-Taxons** If the initial graft for a sample was not originally placed on its taxonomically closest ancestor that exists in PANTHER (and thus there may be a more optimal position to update to) and the graft point is not a leaf node, then ancestral-taxon-nodes of the input species are identified in the set of descendant tree nodes of the graft point. The algorithm prioritizes taxonomic closeness (being a more recent ancestor), followed by total edge distance in selecting an updated graft position (explained in more detail below).
3. **Identify Ancestral-Taxons in Ancestral-Nodes** If no update was performed looking down the tree, the algorithm will determine if the implicit nodes along the parent branch of the initial graft point contain an ancestral node of the input species. If not, the graft point will be updated to the closest taxonomic ancestor node in the set of tree ancestral nodes above the current graft point, or it will be updated to the root if no taxonomic ancestors exist in its tree ancestors (subject to clade blocking constraints if provided, which preventing movement past a given set of clades as described below in the additional details section).
4. **Identify Descendant-Node Ancestral-Taxons Again** After moving upward, the algorithm will repeat step 2 at the updated location. If no further update is made, if the node was updated to the root without it being a direct ancestor, the graft point will be moved back to its initial graft position.
5. **Output** Using the updated graft branch, the set of ortholog predictions are output by determining the set of genes in the grafted PANTHER family which share a speciation node in the tree as their most recent common ancestor and which do not have a horizontal transfer branch on the path between them.

#### Algorithm Priorities

In updating the graft position, OrthoGrafter prioritizes in order: descendant nodes, followed by nodes with the closest taxonomic relationship, followed by those which are closest by edge distance. This gives OrthoGrafter an down-up-down search pattern. First - while taxonomic closeness to the input sequence is desired for the updated graft point, were it given greater weight than edge distance without prioritizing looking down the tree first, long range moves would be significantly more likely when checking the entire tree (as a much more closely related ancestral clade could exist at a distant location in the tree). As TreeGrafter determines its optimal graft location based upon sequence matching, OrthoGrafter assumes smaller moves (thus ending closer to the initial graft point) are more probable, attempting to balance taxonomic closeness with minimal movement from the initial graft position. And smaller moves are more likely when prioritizing looking down the tree first.

When looking down the tree at the descendants of a given node, multiple descendant nodes which exist in a sample’s taxonomic ancestors will only occur as the result of a duplication event. If a closer taxonomic ancestor to the input exists on one branch but not others, it would suggest a higher probability (using only the information contained within the set of PANTHER trees) that the gene may have been lost at some point in evolutionary history along that branch (ie nonfunctionalization). Thus closer taxonomic ancestors beneath a duplication are given preference.

Finally if two descendant nodes share the same degree of taxonomic closeness (ie if the most closely related descendant clade appears two or more times), edge distance is utilized as a tie breaker. The shorter the edge distance along a path based on edge distances, the less divergence from a given node would generally be expected. It is not necessarily true however that the divergence from a node will mirror the divergence from the input sequence (as the input isn’t expected to have the exact sequence of the node). However, were a particular descendant node a significantly better sequence match to the input sequence than the initial graft point, it is again assumed that TreeGrafter would have grafted the input sequence closer to it to start. Thus we assume that the less diverged path to a desired clade is also likely to be less diverged from the input sample. It is perhaps worth noting however, that the direct usage of branch/edge lengths as a measure of divergence could at times be misleading as edge distances are not a direct measure of relatedness. Direct sequence comparison with descendants could be an alternative option, but could suffer from classical long branch attraction when selecting from descendant nodes with longer edge distances, and could negatively impact the speed and size of the algorithm. Edge distance was found to be a convenient measure to estimate divergence while keeping the model relatively light weight.

### Example Placements

**Fig. 1:**
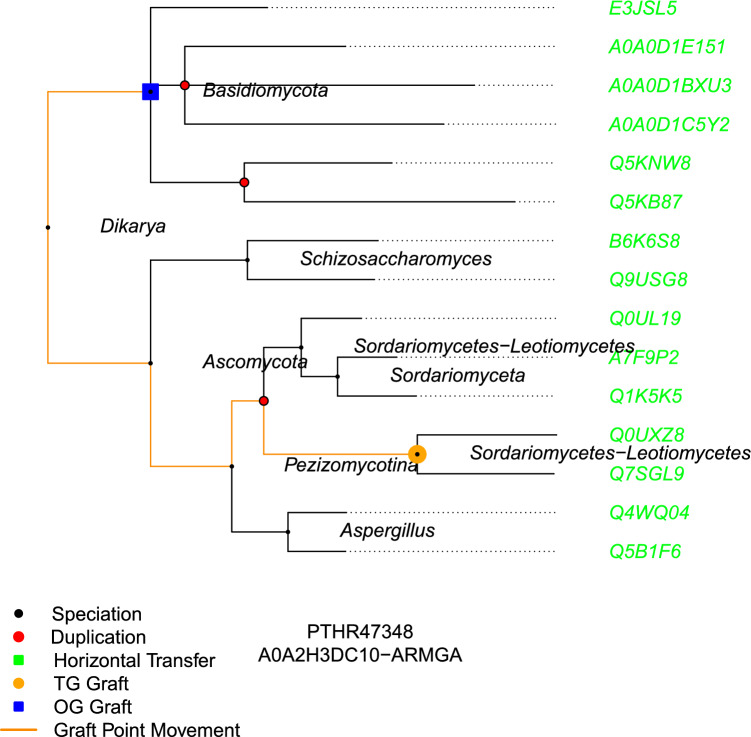
*Example Tree with Sample Graft Updated -* This chart shows the original and updated graft locations for sample protein A0A2H3DC10 (its UniProtKB ID) from the species *Armillaria gallica* (ARMGA) in its grafted tree PTHR47348. The leaf nodes represent genes (labeled with their UniProtKB IDs). The initial graft position is represented by the orange circle with the blue square representing the updated graft location. No child nodes of the initial graft point are taxonomic ancestors so the algorithm moves to the first parent tree node that is a taxonomic ancestor, before looking at the descendant nodes again and moving to its final updated position at clade Basidiomycota, its closest taxonomic ancestor in the tree. Because it moved through a duplication node on its path, the set of orthologs between the initial and final graft positions are not the same. At the updated graft point, all leaf nodes are considered orthologs

An example of the algorithm on a small tree (with a somewhat larger number of node positions moved than average) is shown in Fig. 1. The protein (UniprotKB ID A0A2H3DC10) is placed in the graft tree (PANTHER ID PTHR47348) by TreeGrafter on a node for clade Sordariomycetes-Leotiomycetes. However, the input species is ARMGA (species *Armillaria gallica*, a fungus, ARMGA being its 5 letter mnemonic organism identification code used by UniProt), and the ancestral taxon at which ARMGA and the graft clade diverged was Dikarya. Because PANTHER trees are taxonomically consistent, and since the graft node is not an ancestor of ARMGA, the children will not be either (even were they not leaf nodes as in this case) so the algorithm will not find anything looking down. The algorithm will thus next look up and, finding Dikarya, will jump to the root node in this instance, before looking down once more and identifying Basidiomycota as a closer taxonomic ancestor of ARMGA. The final updated position makes the graft location taxonomically consistent, and because it moved through a duplication node, the set of orthologs (highlighted in green), will contain 3 more orthologs than it would have at its original graft locations, specifically Q1K5k5, A7F9P2, and Q0UL19 would not originally have been predicted as orthologs but paralogs (all genes in a PANTHER tree will be either orthologs, paralogs or xenologs).

**Fig. 2:**
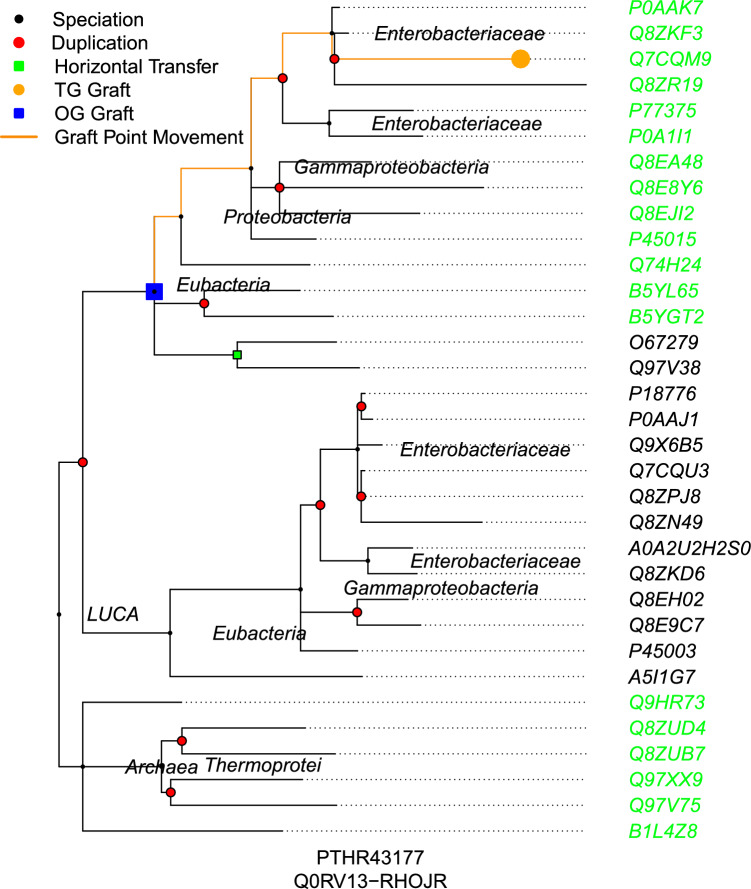
*Example Tree with Sample Graft Updated -* This chart shows the original and updated graft locations for sample protein Q0RV13 (its UniProtKB ID) from the species *Rhodococcus jostii* (RHOJR) in its grafted tree PTHR43177. The leaf nodes represent genes (labeled with their UniProtKB IDs). The initial graft position is represented by the orange circle with the blue square representing the updated graft location. The algorithm updates the graft point to the closest taxonomic ancestor, but since there are no descendant taxonomic ancestors beneath that updated position (highlighted by the blue square), it does not update further. Because it updated the graft position through two duplication nodes, the set of orthologs will be larger than it was for the original graft point. Leaf nodes are highlighted in green for orthologs and black for paralogs/xenologs

A second example (for another small tree) is shown in Figure 2. The input species RHOJR (*Rhodococcus jostii*) is a bacteria, but not an Enterobacteriaceae. The first taxonomic ancestor of RHOJR in the tree is Eubacteria. But there are no other taxonomic ancestors of RHOJR beneath the Eubacteria node, and thus that node becomes its updated graft point. As it passed upward through two duplication nodes, the number of orthologs predicted for this sample will have increased.

Two further examples are provided in Supplementary Figures 1 & 2, demonstrating the basics of how selection is made when going downward through a duplication node.

### Additional Details

Placement in trees is facilitated via the use of the NCBI taxonomy (Schoch et al. 2020). Due to differences in the relational structure of PANTHER and the NCBI (such as ancestor–descendant relationships in PANTHER being siblings in the NCBI taxonomy in a number of cases), as well as the inclusion of a number of PANTHER-specific clades (which do not exist in the NCBI taxonomy), exact mappings between the set of NCBI and PANTHER clades/taxons are not always possible. To handle these cases, where viable, PANTHER-specific clades were assigned to NCBI taxon IDs not otherwise used by PANTHER with approximately equivalent taxonomic locations.

Explicit taxa represent the speciation branch points in PANTHER that exist within a given PANTHER tree. Implicit taxa by comparison represent the set of taxons which exist along branches but are not nodes within the tree. They are the result of missing genes (which would diverge along the branch were they included), either due to gene loss or species not being included in the tree (with only 143 species, many species are not included in PANTHER that would contribute to significantly more branch points). In OrthoGrafter, implicit taxa are used to infer if a sample may have diverged along the branch leading to an ancestral duplication node, and if so to prevent movement upward through said duplication node (as moving through duplication nodes is the major way to alter ortholog predictions). If TreeGrafter predicts a sample to be below a duplication node, and if an implicit node may exist along a branch leading to that duplication node, we consider this situation ambiguous and defer to the prediction made by TreeGrafter.

**Fig. 3:**
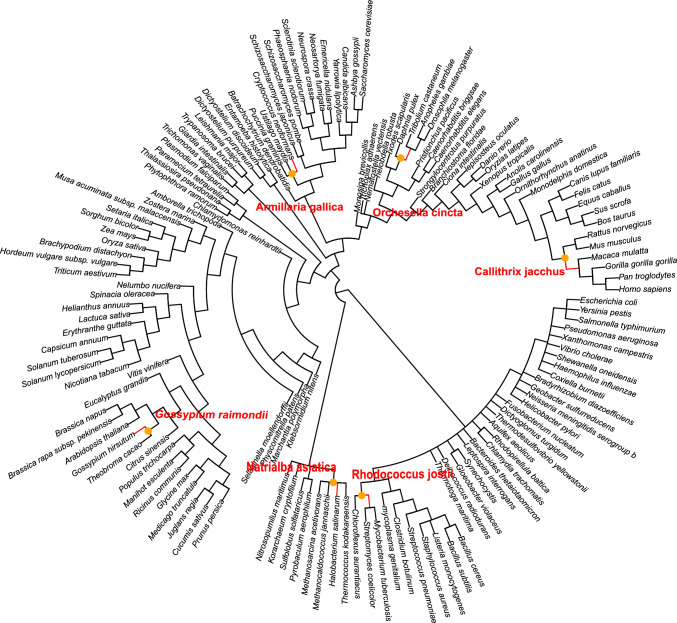
*Test Species Locations in the PANTHER Species Tree -* A visualization of the distribution of test species localized within the PANTHER species tree with the full names of species shown. The orange dots represent the preferential locations for placement by OrthoGrafter (the explicit taxons in the tree). The red branches represent the branches along which the given species diverged (where the implicit taxons they diverge from would be located). Separations between preferred graft locations and the diverging branch  can exist for some species due to the presence of a PANTHER-specific clades lacking implicit NCBI taxon parents

"Blocks" within OrthoGrafter represent a definable list of clades through which graft updates are not allowed to traverse past to a more distant ancestral clade. This can prevent long range movements which would localize the new graft point far from its expected position based on sequence. Moves to a distant branch indicate a large mismatch between the sequence-based graft point from TreeGrafter, and the expected branch point due to species taxonomy, and could indicate issues such as horizontal transfer of the query sequence or gene loss in the current set of PANTHER species. If a horizontal transfer node in PANTHER is on the pathway to a blocked clade (providing evidence for an individual sample that the gene may be the result of a horizontal transfer), OrthoGrafter can output this information. By default we do not allow movement outside of the initial graft’s biological domain (Eukaryota, Archaea or Bacteria).

To illustrate the concept of blocks a bit further, consider Fig. 2. The input sample species RHOJR is not blocked in this example, because it is a bacteria and it does not try to leave the Eubacteria clade. However, if there was a block on Proteobacteria, rather than Eubacteria, RHOJR would be blocked. In such a case because RHOJR is not a Proteobacteria, it would want to move up past Proteobacteria, which would not be allowed if it were a blocking clade. Alternatively, if the input sample had been NATA1, an Archaea, Eubacteria would not have been one of its ancestors, and NATA1 would have been blocked from moving up past Eubacteria to LUCA, so it would not have been able to update its graft position, even though there are Archea elsewhere in the tree (Archea whose genes would potentially have been distantly related however based on the initial graft location).

Finally, if the initial graft point is placed at the root node, this might indicate that TreeGrafter recognizes the sample as belonging to part of a larger super tree family (as TreeGrafter is effectively grafting to the parent branch of the graft node). We treat such instances as ambiguous and do not update the graft position in these cases.

#### Algorithm Performance and Outputs

It is worth noting that OrthoGrafter has been written with the attention of being efficient when running large sample sets, utilizing precomputed datasets for rapid identification of taxonomic and orthologous relationships. Data on the programs performance is shown in Supplementary Table 2.

OrthoGrafter can additionally provide other potentially useful information, such as the sets of paralogs and xenologs from PANTHER trees, the least diverged ortholog, the graft point distance moved as well as other information of interest. And while all samples to OrthoGrafter are updated independently and OrthoGrafter does not construct new trees, orthologous relationships between input samples can be predicted via a separate check.

### Testing of the Graft Point Updating Procedure

#### Testing Method

To test the result of OrthoGrafter’s graft point updates and whether they improve the orthology inferences, we constructed a test set of orthologs from the OMA database for comparison (Altenhoff et al. 2024b). OMA was selected because it is a popular ortholog database containing a large list of orthologs from many species, including all the species in PANTHER, and can thus be used for benchmarking. From published benchmarks of orthology methods (Nevers et al. 2022), we expected there to be considerable discrepancy between OMA and PANTHER orthologs; the primary source of this discrepancy is that OMA tends to have higher specificity but lower sensitivity. Therefore, for our test, we do not necessarily expect a high level of agreement between OrthoGrafter and OMA, and expect overall a high number of apparent “false positives.” These may not actually represent incorrect orthologs from OrthoGrafter, but rather reflect the emphasis on specificity in OMA. We therefore focus on the relative performance of OrthoGrafter with, and without, the updating reconciliation procedure.

**Table 1:** Experimental testing set data

Species	Sample Count	Ancestral Clade	Species Name
ARMGA	7339	Fungi	*Armillaria gallica*
CALJA	20,177	Mammalia	*Callithrix jacchus*
GOSRA	32,964	Viridiplantae	*Gossypium raimondii*
NATA1	2156	Archaea	*Natrialba asiatica*
ORCCI	9246	Arthropoda	*Orchesella cincta*
RHOJR	5259	Eubacteria	*Rhodococcus jostii*
Total	77,141		

This table lists the names of species (as well as their 5 letter mnemonic organism identification codes) used for the testing set, their ancestral clade group and the number of samples (ie proteins per species) used in the testing set for comparisons between OrthoGrafter’s and TreeGrafter’s graft points

Sets of proteins taken from 6 distinct extant species representing diverse ancestral clades were chosen for comparison. The species were chosen such that each species is both a member of a chosen distinct ancestral clade which is not in PANTHER, as well as having the largest number of orthologous pairs in the set of OMA pairs where the other member of the pair is in PANTHER. The set of chosen parental clades, the species selected and the number of protein samples for each species is shown in Table 1.

The distribution of the species among the set of all PANTHER clades, their optimal taxonomically consistent graft points for OrthoGrafter and the branches along which they diverged is visually represented in Fig. 3. TreeGrafter locations for testing were obtained using precomputed graft points determined by Uniprot via InterProScan (Blum et al. 2024), this data set’s download location is provided in Supplementary Table 1.

For testing purposes the set of OMA ortholog pairs were used to represent truth values for benchmarking. Positives are those ortholog pairs predicted by OrthoGrafter. True positives were considered to be any pair of orthologs predicted by OrthoGrafter, which were also in the set of OMA pairs. False positives were taken as the set of ortholog pairs from OrthoGrafter which do not exist in the set of OMA pairs but for which there was an ID mapping to Uniprot for both proteins in each pair (OMA pairs are initially provided using their own ID type with a mapping list between OMA IDs and Uniprot IDs, any protein in PANTHER was only taken as relevant if it has a mapping from OMA to Uniprot).

Negatives represent the pairs predicted as paralogs or xenologs by OrthoGrafter. True negatives being those which again have an OMA ID mapping, but are not listed as orthologs by OMA, and false negatives which OMA lists as orthologs. A list of these explanations is shown in Table 2.

**Table 2:** The definitions for the elements of the confusion matrix used for making comparisons to OMA ortholog predictions. In all cases mappings must exist from OMA IDs to Uniprot IDs

Term	Explanation
TP (True Positive)	Orthologs identified by OrthoGrafter and contained in OMA
FP (False Positive)	Orthologs identified by OrthoGrafter which do not exist in the set of OMA pairs
TN (True Negative)	Xenologs or Paralogs identified by OrthoGrafter that are not in the set of OMA pairs
FN (False Negative)	Xenologs or Paralogs identified by OrthoGrafter that are in the set of OMA pairs

Data sets used for this experiment are listed in Supplementary Table 1.

## Results

**Table 3:** Comparison of Average Benchmark Scores on OMA Data for Initial and Updated Graft Positions—Initial graft positions were obtained from TreeGrafter, with the updated positions from OrthoGrafter

Metric	OrthoGrafter	TreeGrafter
True Positive Rate (TPR)	0.847	0.811
False Positive Rate (FPR)	0.494	0.470
F1 Score	0.426	0.421
Matthews Correlation Coefficient (MCC)	0.289	0.273

Rates were obtained for individual samples based on the number of proteins in their grafted family which are considered orthologs (and also exist within the set of OMA IDs that map to UniProt). The scores here reflect the average values of the metrics for the individual samples in their associated trees

A total of 77,141 proteins with precomputed TreeGrafter graft positions were obtained for the given 6 species with placements in a total of 12,567 unique PANTHER trees. 34,281 (44%) of the proteins had their graft position updated by OrthoGrafter. Of those however only 31,387 samples were found to have at least one ortholog in the set of OMA pairs. Within this set the median number of nodes moved was 1 and the median edge distance moved was 0.13 (2.2 and 0.50 for the mean values, respectively) with approximately 1.3% of samples blocked from moving outside of their domains. A chart showing the frequency of movements by the number of nodes moved is shown in Supplementary Figure 3.

**Table 4:** Difference in average ortholog results by species—this table shows the difference in the average sample F1 scores for the separate test species for OrthoGrafter (OG) and TreeGrafter (TG), as well as the total number of samples for each species, how many were updated and the fraction updated

input	OG F1	TG F1	# Updated	# Total Samples	Fraction Updated
ARMGA	0.334	0.336	3009	7339	0.41
CALJA	0.464	0.461	7067	20,177	0.35
GOSRA	0.463	0.456	12,436	32,964	0.38
NATA1	0.334	0.308	250	2156	0.12
ORCCI	0.369	0.364	5049	9246	0.55
RHOJR	0.384	0.378	3576	5259	0.68

Average statistics measuring the impact of the graft position update on the inferred orthologs are shown in Table 3. As the updates generally resulted in small moves, we might expect a small effect on the predicted orthologs, which matches what is observed. The most relevant score is likely the MCC score (The Matthew’s correlation coefficient, see formula below), which takes into account all values in the confusion matrix, with a correlation value from -1 to 1 (1 representing perfect prediction). For the average of the MCC scores for the input samples we observe a small but positive change of 0.015, the change in the F1 score is smaller at 0.05, but it is in the correct direction.

$$\begin{aligned} \text {MCC} = \frac{(TP \times TN) - (FP \times FN)}{\sqrt{(TP + FP)(TP + FN)(TN + FP)(TN + FN)}} \end{aligned}$$

To help put these scores into context, random updates were also performed using the same set of samples. Five new sets using the same 77,141 samples were created, and random values for the number of nodes moved from the initial TreeGrafter graft point were assigned to each sample by drawing with replacement from the distribution of nodes moved for the OrthoGrafter set (as shown in Supplementary Figure 3). The difference between the scores found using ortholog sets with random moves and those found using TreeGrafter’s initial graft points for the five sets were found to have a mean of $-$0.020 (SD 0.001) for MCC (compared to the OrthoGrafter updates with a positive 0.015 MCC difference). And for mean of the difference for the F1 score was $-$0.009 (0.0002 SD) versus the 0.005 change with OrthoGrafter. While these differences are small they are fairly consistent and offer context that overall changes to the scores using OMA as a benchmark tend to be small, and that OrthoGrafter shows a comparable degree of improvement over TreeGrafter graft points, as TreeGrafter shows over graft points randomly assigned nearby.

**Fig. 4:**
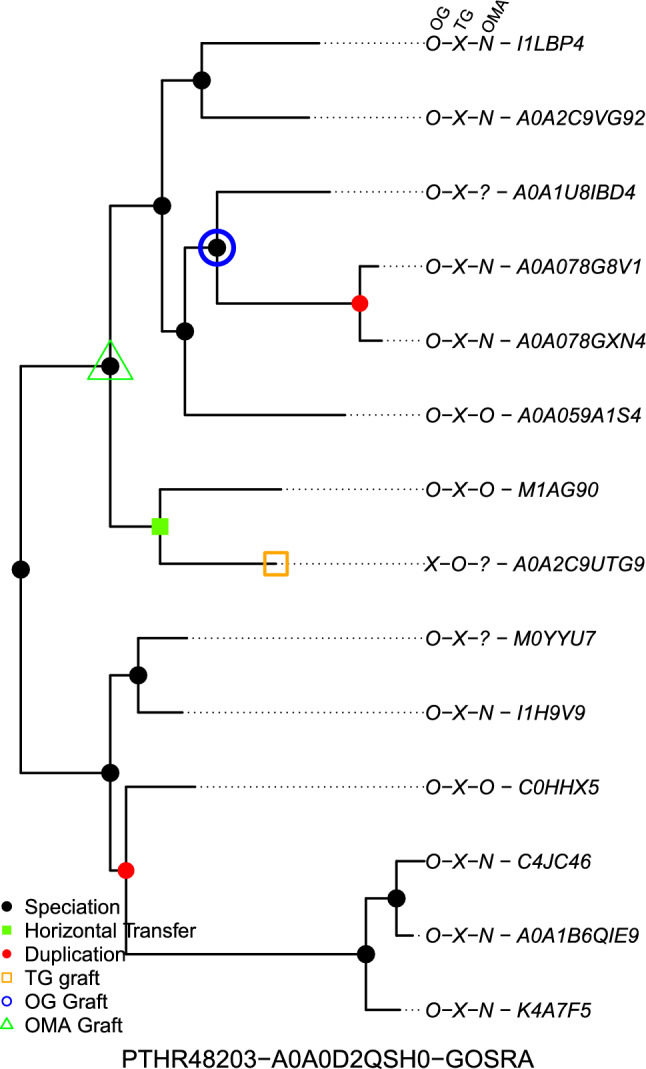
*Annotations Comparison of OrthoGrafter with TreeGrafter and OMA Pairs -* A PANTHER gene tree (ID PTHR48203) for sample A0A0D2QSH0 (UniProt ID) which is a member of species *Gossypium raimondii* (GOSRA). The predictions for OrthoGrafter (OG), TreeGrafter (TG) and OMA are shown along with the leaf UniProtKB IDs, (O=Ortholog, P=Paralog, X=Xenolog, N=Not Ortholog, ?=Unknown. Unknown means that OMA lacks an OMA ID that maps to UniProt for the given protein). The initial TreeGrafter graft point (orange circle) and updated graft from OrthoGrafter (blue square) are shown as well as the green triangle representing the closest position based on edge distance to the initial graft point which maximizes the number of predicted orthologs labeled as true positives for the OMA set. For this example, OrthoGrafter’s predictions show greater agreement with OMA’s predictions

The increased true and false positive rates reflect the tendency of the updated graft position to predict a higher number of orthologs (Which can be the result of moving up past a duplication node within a tree, thus increasing the total number of predicted orthologs while reducing the number of paralogs). By comparison moving down the tree is likely to reduce the number of orthologs, but likely by a smaller amount (going up tends to give access to a larger subtree that are predicted as orthologs, whereas going down would "cut off" a smaller subtree). Upwards movement thus enables larger increases in the number of orthologs predicted than downwards movement reduces predictions. The sample graft updates also showed almost 12,000 upward movements through duplication nodes but only around 4000 samples with downward movement through a duplication node. Hence higher TPR and FPR rates.

Due to the fact that the different test species had significantly different numbers of protein-coding genes (with GOSRA representing almost half the total number of sequences in the entire set) scores were also broken down individually by species as shown in Table 4. There are substantial differences in the number of updates and their impact on the F1 score dependent on species, highlighting that the impact of the updating procedure in OrthoGrafter can be species dependent.

Finally an example output is shown in Figure 4 for sample A0A0D2QSH0. The initial graft point is along a horizontal transfer branch, with all other leaf nodes in tree being xenologs of that graft position. In this example TreeGrafter shows a fair bit less agreement than OrthoGrafter with OMA’s ortholog predictions. However, it is not possible to obtain complete agreement with OMA, as any position in the tree would give more incorrect predictions (generally false positives) than correct ones. This might be partially explained by OMA’s lower sensitivity compared to PANTHER on benchmarking tests (Altenhoff et al. 2016), OMA has an apparent tendency to exclude orthologs that are highly diverged in sequence (having long branches), leading to false positives when using a somewhat more permissive method such as PANTHER.

While overall the results do seem to suggest a small improvement for OrthoGrafter compared to TreeGrafter, this is not true for every tree, Supplementary Figures 4 & 5 show additional example of updates on trees, but for cases where the results show less agreement with OMA than the initial graft point.

## Discussion

Overall by itself TreeGrafter’s placement within the PANTHER trees shows a high level of taxonomic accuracy in its placement in the PANTHER trees, with most sequences (56%) found not to benefit from updates to their graft location. Even for the cases where updates were made, most were moved a fairly small distance in terms of both number of branches, and total branch length. However, we did observe a small but noticeable improvement in consistency with an independent orthology method (OMA) based on the change in the MCC score that were obtained from our updated and more taxonomically consistent graft positions, and ultimately the placement in the taxonomic trees should be taxonomically consistent if one wants to obtain the correct set of orthologs.

There still exist a number of remaining challenges with the current implementation of OrthoGrafter described here. As discussed above, the mapping between the NCBI taxa and those in PANTHER are not exact, due to the presence of additional clades in the species tree used by PANTHER, which can make the optimal graft location to reconcile a sample unclear in some cases. PANTHER’s calculation of total branch distance also has limitations, with low-confidence branches arbitrarily assigned in PANTHER to have a length of 2 substitutions/site.

Moving down (leafward) past a duplication node also comes with limitations. Moving through duplication nodes (or through horizontal transfer branches) is the only type of movement which will alter the predicted ortholog sets, but when moving down past a duplication node, the question of what branch to select when two or more might be valid can have an appreciable impact on the final set of orthologs predicted. The easiest solution to this ambiguity and the one used for OrthoGrafter is to select the closest node based on ancestral proximity and edge distance. A deeper consideration of this problem and how to better measure divergence between nodes and how to determine the most probable branch could lead to further improved results.

## Supplementary Information

Below is the link to the electronic supplementary material.

Electronic supplementary material 1 (PDF 463 kb)
