# Supplementary material for: OrthoGrafter: Rapid Identification of Orthologs from Precomputed Placement in Phylogenetic Trees
Source: J Mol Evol. 2025 Nov 22;93(6):820–9. doi: 10.1007/s00239-025-10279-z (PMC12756373; doi:10.1007/s00239-025-10279-z)
Supplement: Supplementary file 1 — Electronic supplementary material 1 (PDF 463 kb) [file 239_2025_10279_MOESM1_ESM.pdf]

OrthoGrafter - Rapid Identification of Orthologs from Precomputed Placement in Phylogenetic Trees
Supplementary Data

Authors : Christopher M. Williams, Paul D. Thomas
University of Southern California,
Department of Population and Public Health Sciences,
Los Angeles, California, USA
will662@usc.edu

October 5th, 2025

Supplemental Data

Description	Details / Source
PANTHER (ver 17)<br>Note:	https://data.pantherdb.org/ftp/downloads/TreeGrafter<br>Modified Newick Trees w/ extended NHX data
NCBI taxonomy (rel 2022-08-18)	https://obofoundry.org/ontology/ncbitaxon.html
OMA pairs (2024-07)	https://omabrowser.org/All.Jul2024/oma-pairs.txt.gz
Precomputed TreeGrafter Locations<br>Note:	https://www.ebi.ac.uk/interpro/download/<br>File named "match_complete.xml"

Table 1 Data sources and versions used for tree placement and taxonomy ontology

Max Memory (GB)	Sample Count	Time (sec)	Trees	Species	Sec/Sample
1.62	77141	8000	12567	6	0.1
1.22	1000	283	796	1	0.28
0.80	100	55	94	1	0.55
0.46	10	31	7	1	3.1
0.41	1	26	1	1	26

Table 2 *Table of Benchmarks for Algorithm Speed* - A table showing the maximum memory used for sets containing different number of samples and the corresponding total number of trees those samples were grafted into, and how many different species were contained in the sample set. The final column gives the number of seconds taken per sample on average. All tests were run on a laptop with an AMD 7840HS processor with a base clock of 3.8 Ghz, the algorithm currently is single threaded.

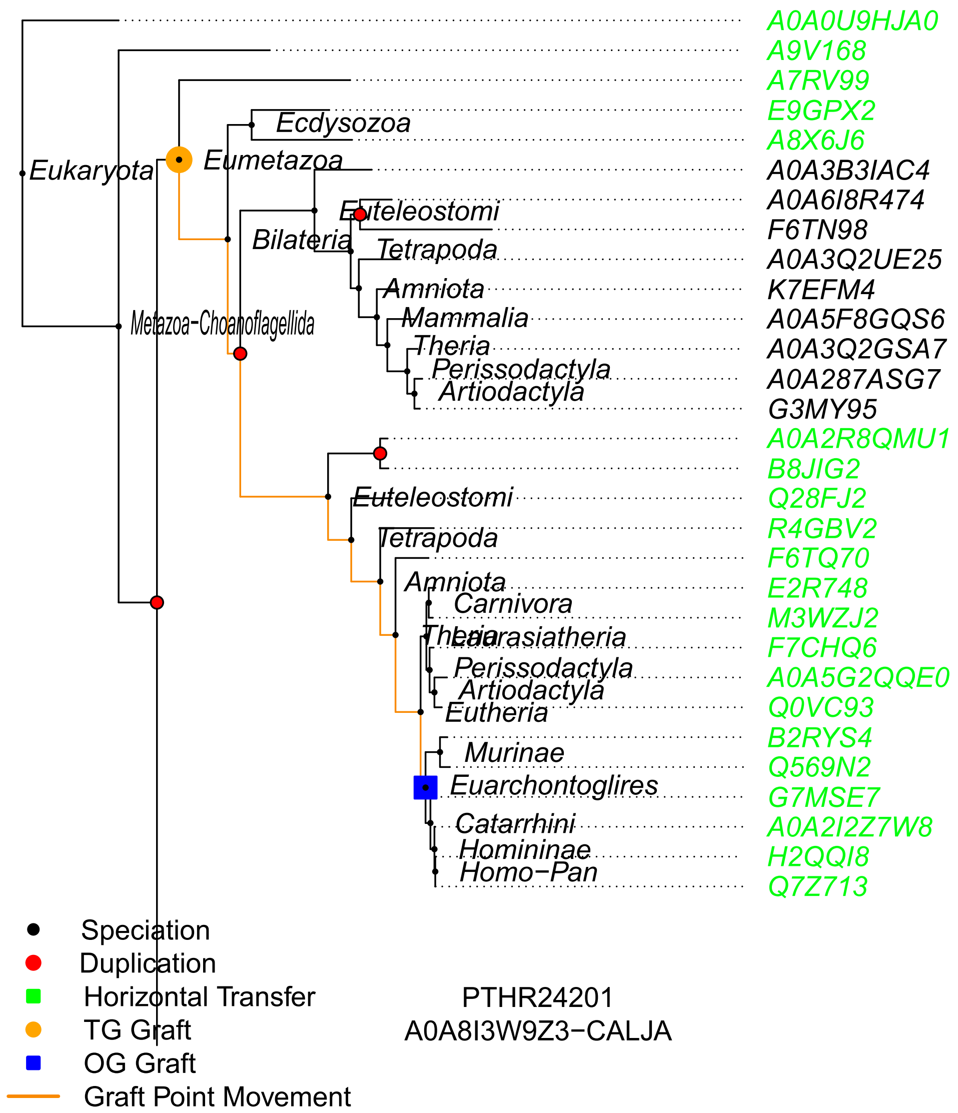

Fig. 1 *A Tree Showcasing Disagreement with OMA # 2 -* This tree shows sample A0A8I3W9Z3 (UniProtKB ID), a protein for the species CALJA, grafted in a subtree of PANTHER tree PTHR24201. When updating the position of the graft point, the algorithm first looks down the tree. In this case it finds two branches of a duplication node with taxonomic ancestors of CALJA. However the closest taxonomic ancestor in the descendant tree nodes is Euarchontoglires, which is only on one branch of the duplication node. The algorithm thus selects this node to update the graft point to. Because the update moved through a duplication node, this choice will change the predicted ortholog set.

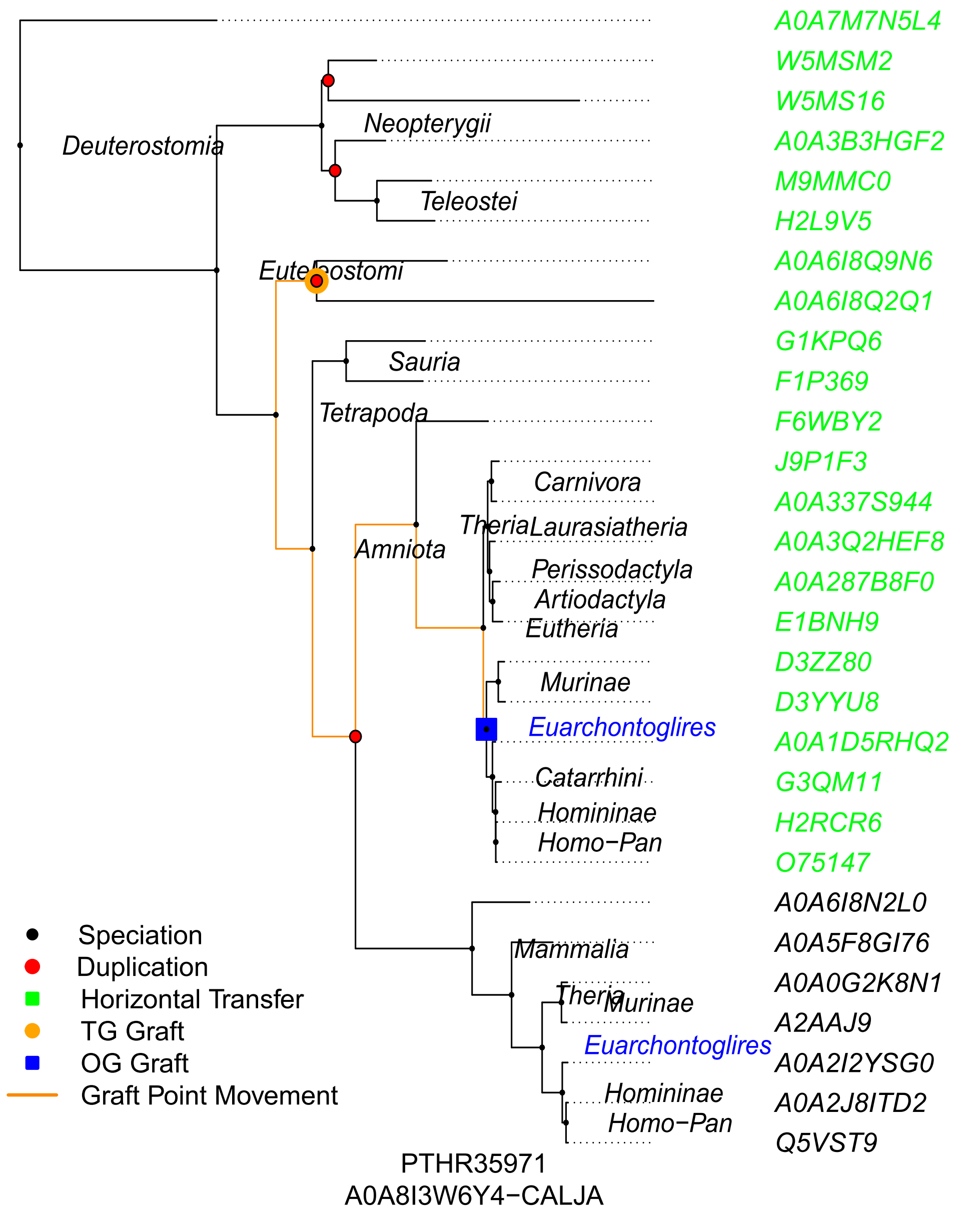

Fig. 2 *A Tree Showcasing Disagreement with OMA # 2 -* This tree shows sample A0A8I3W6Y4 (UniProtKB ID), a protein for species CALJA, grafted in PANTHER tree PTHR35971. When updating the position of the graft point, the algorithm first finds no descendant nodes of the initial graft point as taxonomic ancestors. It next looks up the tree and finds Tetrapoda is a taxonomic ancestor (the closest tree node that is a taxonomic ancestor will also be the closest taxonomic ancestor because PANTHER trees are reconciled). It will update to Tetrapoda and then look down the tree once more. In this case it finds two Euarchontoglires nodes (highlighted in blue) below different branches of the duplication node as its closest ancestors. The algorithm will prioritize the least diverged Euarchontoglires node from the initial graft position predicted by TreeGrafter, which is determined based on the smallest branch distance. This choice will impact the set of orthologs predicted.

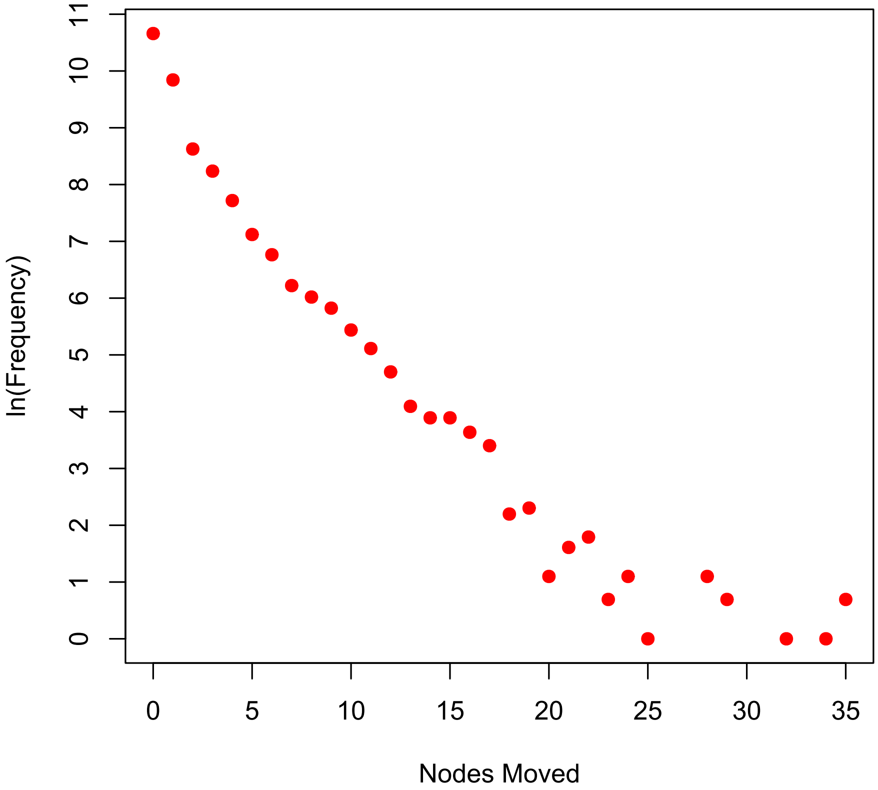

Fig. 3 *Natural Log Plot of the Number of Nodes Moved When Updating from Initial Graft Point* - This shows a plot of the natural log of the number of samples based upon the number of nodes those samples moved when updating the graft point, a decreasing linear trend can be observed.

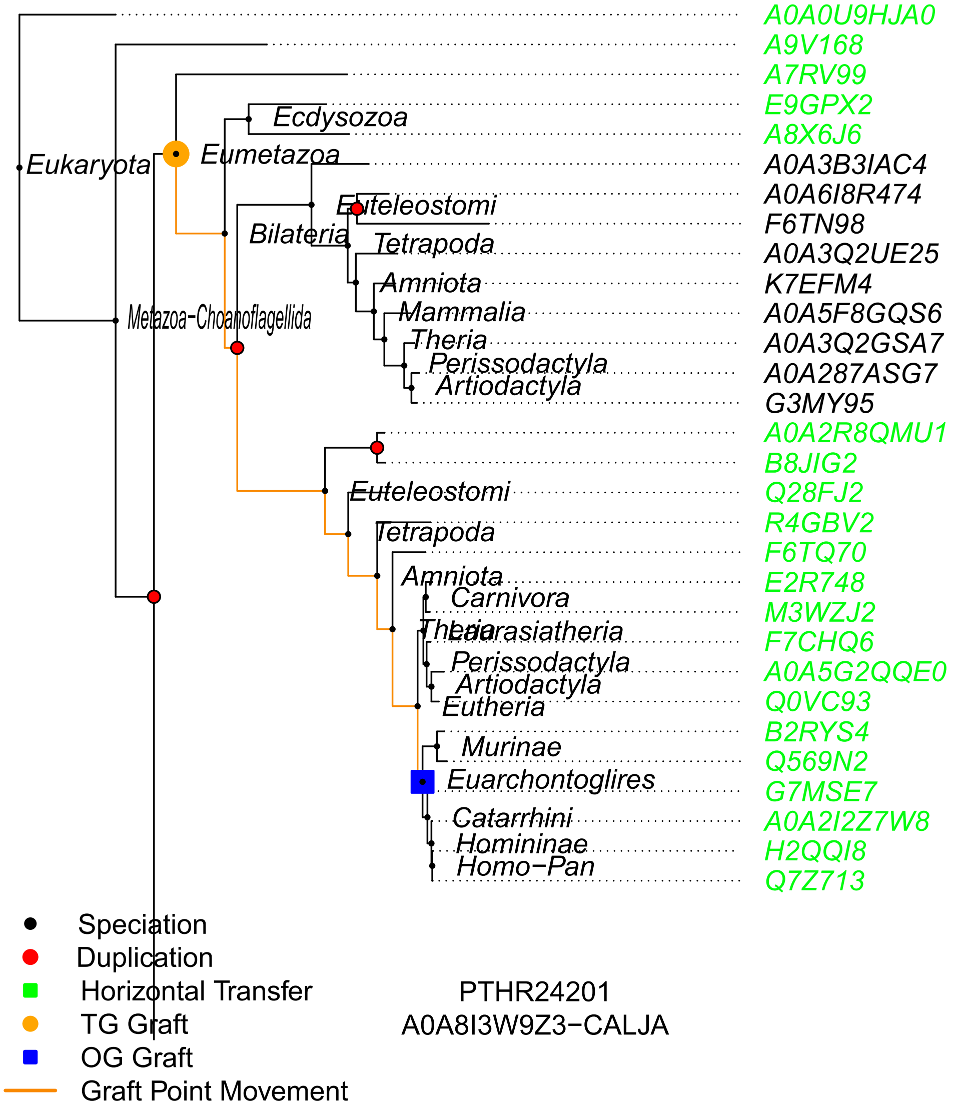

Fig. 4 *A Tree Showcasing Disagreement with OMA* - This tree shows sample A0A2H3DC10 (UniProtKB ID) a protein for the species ARMGA, grafted in PANTHER tree PTHR47348. The predictions for OrthoGrafter (OG), TreeGrafter (TG) and OMA are shown along with the leaf UniProtKB IDs, (O=Ortholog, P=Paralog, X=Xenolog, N=Not Ortholog (or negatives)). There is only one ortholog pair listed by OMA for the input sample and a number of Ns, with the optimal position to maximize the score when benchmarking against OMA is the graft point found by TreeGrafter (which predicts one more negative in line with OMA than the updated graft point). This is the same sample shown as an example in the main paper in Figure 1, and as discussed, TreeGrafter graft position is not taxonomically consistent, however. Thus while TreeGrafter matches OMA better, neither TreeGrafter nor OMA is consistent with the PANTHER tree here. No matter where one graphs it is not possible to obtain full consistency with OMA.

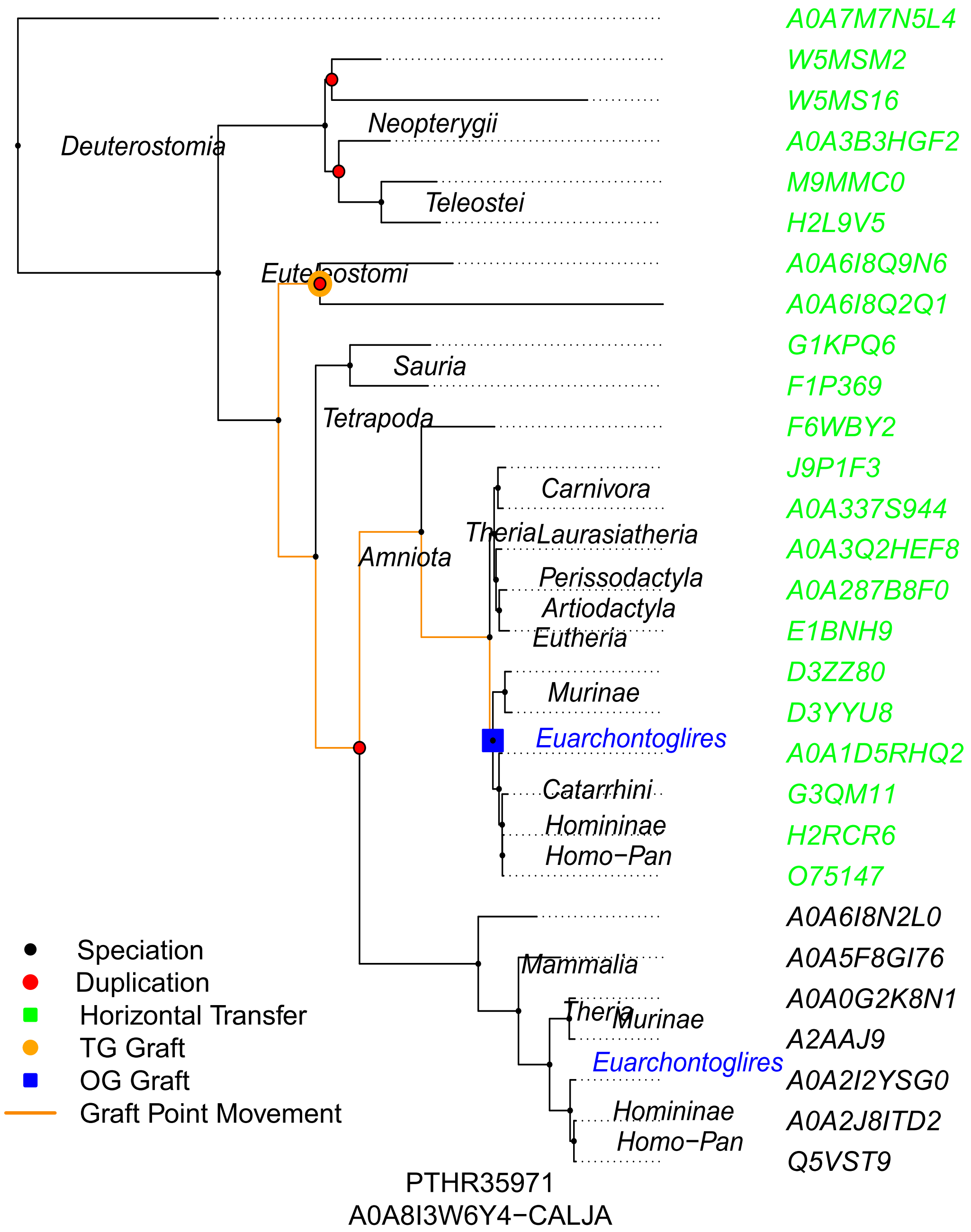

Fig. 5 *A Tree Showcasing Disagreement with OMA # 2 -* This tree shows sample A0A0D2QZS0 (UniProtKB ID) a protein for the species GOSRA, grafted in PANTHER tree PTHR37611. The predictions for OrthoGrafter (OG), TreeGrafter (TG) and OMA are shown along with the leaf UniProtKB IDs, (O=Ortholog, P=Paralog, X=Xenolog, N=Not Ortholog). There are only two true positives (orthologs predicted by OMA) in this tree, one of which shares its most recent common ancestor with both the initial and final graft point, the other exists directly across a duplication node. In this case it is an open question when dealing with moving a graft point directly up past a duplication node to the next closest speciation node, whether or not the sample diverged before or after the duplication. Because TreeGrafter is often slightly off, a small move of this sort would seem to be a something of a guess, where different samples may have different results.
